# Delegated disabling affects in partnership

**DOI:** 10.3389/fsoc.2025.1422337

**Published:** 2025-02-27

**Authors:** Judith Tröndle

**Affiliations:** Department of Education and Social Work, Institute for Social Research and Interventions (ISI), Université du Luxembourg, Esch-sur-Alzette, Luxembourg

**Keywords:** disability, parenting, couples, affect, subjectivation, gender, emotion

## Abstract

The social and cultural understanding of disability has indicated that it is primarily a consequence of attributional processes, idealized and generalized conceptions of ability, and structural discrimination. Assuming the validity of these conceptualizations, the focus shifts to relational dynamics that determine how and if disability is ‘felt.’ This study explores this relationality in the context of couples parenting a child with disabilities. Intersections of gender and disability associated with self-positioning as ‘special parents’ include specific affective couple arrangements. This study reports on a qualitative study using in-depth interviews with couples who were interviewed first together and then individually. The results indicate a subjectivation of couples as ‘special parents,’ which is difficult to reject and includes affective aspects as well as gendered inequalities in care. Disabling affects are delegated to and felt by the female partner, leading to *affective inequalities* in the partnership. The couple positions the mother as the one who ‘suffers,’ which is part of a well-known affective repertoire that is implied by ableism to feel. The theoretical implications of these empirical results will be discussed as twofold: first, as an entry point to understanding *disability via affection*—how to be affected by disability along intersected cultural attributions; and second, as a suggestion to bridge cognitive and behavioral approaches to emotion by elaborating on how *disabling affects become felt and enacted in subjectivation and relation*.

## 1 Disabling affects felt in subjectivation

One may state that disability studies contribute to the decentering of an individualized, relatively autonomous subject. With a conceptualization of disability as a matter of inequality in social structure on the one hand (Hughes and Paterson, 1997) and in turning toward an understanding of (dis-)abled subjects as cultural appearances on the other (e.g., Waldschmidt, 2017a; Goodley, 2014). Studies on (dis-)abling subject formation deconstruct essentialist and medicalized attributions to individuals. They illuminate the historical embedding of knowledge and power production around disability and the (re-)production of difference through othering. Furthermore, they facilitate linking the institutionalization of discursive knowledge on disability to understand (missing) actions or social self-positioning (e.g., Pfahl, 2011; Karim, 2021; Buchner, 2018; Czedik and Pfahl, 2020). However, an aspect that is largely overlooked is *affective formation as part of subjectivation*. It is suggested in this study that the “productive power” in a Foucauldian sense (Foucault, 1989 a. o.), the “interpellation” (Althusser, 1977), or the subjection of “The Psychic Life of Power” (Butler, 1997) does not end with social positioning. It also generates frames of desire, perception, and affect. As Traue and Pfahl (2022) put it, *“Subjectivation, we might say, requires an activity from the individual, which is not simply a ‘mirroring’ of expectations but an affective action through which being-affected, relationality, and valuation ‘become felt.’”* (ibid. 34). Since empirical and theoretical elaborations on (dis-)abling formations of subjects’ affectivity are still largely missing, the study contributes to this perspective.

Empirically, I report on a study on heterosexual couples parenting a child with disabilities (Tröndle, 2022a). The position of parents has been critically discussed by the disability rights movement with regard to power relations in care (Carey et al., 2020; Goodley and McLaughlin, 2008; Ryan and Runswick-Cole, 2008). This study takes this discussion into account and understands parental ambivalences as part of the disabling process of (socially speaking) becoming ‘special parents.’ This study integrates research on subjectivation with perspectives from the sociology of knowledge, thereby building upon extant work in the domain of empirical research on subjectivization (e.g., Traue, 2010; Pfahl, 2011; Schürmann, 2013). The phenomenological-interpretative approach facilitates a comprehensive reconstruction of emotional meaning-making at the level of text and performative interaction (in interview transcripts). In the reconstruction of emotional meaning-making, the manner in which emotional content is expressed, the timing of its articulation, and the addressee of this expression are of significance. Although the majority of research on parents of children with disabilities has focused on mothers, the present study included data from couples and individual interviews with both mothers and fathers. This study elucidates the affective dimension of this process of becoming. Following an overview of the conceptual framework, the study results on affectivity are presented. Based on the results, I suggest an understanding of disabling affect as part of a process of subjectivation and discuss how affection and its rejection, contribute to gendered inequalities in the couple. Furthermore, this will be conceptualized as a component of “emotional inequality” (Illouz, 2012, 2008, 2007) and as *affective activity*. In the last part of this study, this interpretation is discussed in light of recent theorizations in the field of sociology of emotions and disability studies. It is argued that, albeit from different entry points, both research fields share the aim of approaching the interrelations of materiality, bodies, and cultural frames of interpretation.

### 1.1 Disabling affect

What can be considered a ‘disabling affect?’ There are certainly several answers to this question, ranging from others’ affection and affection toward othering to othered affection. Despite contributions toward an understanding of disabling affect (e.g., Wechuli, 2024, 2023a, 2023b) and affect and feeling from the perspective of disability studies (e.g., Goodley et al., 2018; Jackson, 2021; Liddiard, 2014; Runswick-Cole, 2013; Hughes, 2012), the systematic connection of these concepts and a consistent theorization of their forms of appearance in processes of subjectivation are still missing. However, the question of how disability is felt, or how this affection can be rejected, remains unanswered, although it can be expected to add important perspectives to disability studies. A concept of disabling affect, I argue, potentially mitigates theoretical divisions of bodies (impairment), social-material structure (social model), culture (cultural model, ableism, othering), and materiality (barriers, assistive devices). Focusing on affectivity offers new perspectives on the interplay of social structure, cultural interpretation of (dis-)ability, somatic sensation, and experience. Gregg and Seigworth (2010, p. 3) put it: “With affect, a body is as much outside itself as in itself —webbed in its relations—until ultimately such firm distinctions cease to matter.” The ‘muddy’ position of affect between body and mind has been approached through a multitude of interdisciplinary conceptualizations. Hence, empirically approaching the disabling affect level is not evident, nor is it an answer to the theoretical gaps in the field *per se*. This requires conceptualization of affect and disability in empirical approaches. I will refer to affect when approaching the empirical phenomenon of being affected by disabling interpellation. I also recognize the variety of terminologies in the field because they include inseparable aspects. The specific potential of orienting attention towards affect in subjectivation addresses existing theoretical divisions between bodies, social structure, and culture. In the words of Sarah Ahmed, affect “sticks… sustains or preserves the connection between ideas, values, and objects” (Ahmed, 2010a, p. 29). Affect becomes felt by subjects and is, at the same time, part of historically specific knowledge formation that contributes to subject formation. Additionally, emotion is used in this study as an umbrella term with regard to specific concepts that I consider helpful in approaching disabling affect, namely the theorization as “embodied emotions” (Hufendiek, 2016) and the suggestion of “emotional inequality” (Illouz, 2012, p. 107).

Based on theories of enactment and embodiment, Hufendiek (2018, 2016, 2014) suggests an approach that allows a general location of affect between cognition, body, and the normative structured environment. She argues that “affordances allow for an enactive account of emotions, externalized social norms allow for an embedded account of emotions, and embodied reactions constitute the skillful knowledge through which we grasp the social rules and norms that form emotional content. Taken together, this leaves us with a picture of emotional reactions that do not exist in the head alone but are rather constituted by the structured environment and the skillful embodied agent” (Hufendiek, 2014, p. 377). This theoretical localization of affect as embedded and embodied allows for the connection of emotion to the structured environment without rejecting the idea of a skillful agent toward social norms.

The concept of “emotional inequality” introduced by Illouz (2012, 2008, 2007) refers to a historicization of emotion that sheds light on capitalist and gendered orders of emotion. The seminal study by Arlie Russell Hochschild also represents an important point of reference in this context. In her study, she develops the concept of “emotion work” (Hochschild, 1979, p. 572), which she also discusses as “emotion management” (Hochschild, 2012 [1983], p. 7) or, most prominently, as “emotional labor” (ibid.). She defines emotional labor as “[…] the management of feeling to create a publicly observable facial and bodily display; emotional labor is sold for a wage and therefore has *exchange value* […]” (ibid. emphasis in original). Moreover, she states that she employs the terms “emotion work” and “emotion management” synonymously to “[…] refer to these same acts done in a private context where they have *use value*” (ibid. emphasis in original; see also Hochschild, 2012 [1983]). This empirical analysis can be described as a groundbreaking achievement in the marking of class- and gender-specific usage of emotions and their physically and visibly expressed forms. In the case of the study this research reports on, however, the aspect of emotional use is not the focus. In the context of the study results, the couple-interactive attributions of emotional experience do not appear to be a value that is used. Instead, emotion becomes evident at the couple level, where gendered attributions are reproduced. It is negotiated as belonging to one of the two partners, which manifests gendered inequalities between the partners. Furthermore, Hochschild posits that “By ‘emotion work’ I refer to the act of trying to change in degree or quality an emotion or feeling” (Hochschild, 1979, p. 561). The act of changing emotions is not applicable in this case. Instead, as will be discussed later, subjectivation processes are pivotal. These do not result in an act of feeling differently; rather, they merely permit specific “affective repertoires” (von Poser et al., 2019). These, it can be argued, are shaped by gendered and disabling norms of care and heterosexual partnership. At last, Eva Illouz’s notion of emotional inequality appears to be particularly pertinent here, given that it was developed with a view toward elucidating the historical and affective transformations occurring in romantic relationships. While some studies in the field of disability studies have already shed light on gendered care in parenting (e.g., Traustadóttir, 2006, 1995, 1991; Goodley and McLaughlin, 2008; McLaughlin et al., 2008; Ryan and Runswick-Cole, 2008), studies on affects in parenting a child marked as disabled are still largely missing. This is mostly due to restraints toward research of care relations in disability studies (exception, e.g., Jackson, 2021). Hence, this study elaborates on the affect around intersected disabling and gendered interpellation that couples parenting a child with disabilities confront. In their responsibility for, and the literal bodily and emotional closeness to, their othered child, include their experiences of othering and discrimination within ableist societies. Furthermore, the couple as a—still—romantic, heteronormative construction includes gendered inequalities. It also comes along with a specific “set of affects,” attached to cultural interpretations and expectations. These “affective shimmers” (Gregg and Seigworth, 2010) were reconstructed in the interpretive analysis of the narration and interaction in and through language. With this approach, disabling affects are considered as appearances in couple relationships—more precisely, as *felt parts* of a specific subject formation as parents of a child with disabilities. With the example of parenting couples, becoming subjected as “special parents,” the study relies heavily on the explanatory framework of a social and cultural understanding of disability (Waldschmidt, 2017a, 2017b; Waldschmidt and Schneider, 2012; Mik-Meyer, 2016; Oliver, 2009; Campbell, 2008; Snyder and Mitchell, 2006; Hughes and Paterson, 1997 a. o.). Furthermore, it relies on theoretical and empirical work on subjectivation studies (specifically Bosančić et al., 2022; Traue and Pfahl, 2022; Ricken, 2013; Pfahl, 2011; Meißner, 2010; Butler, 1997).

Empirically, this study reports on co-constructed narration and the interaction of couples as entry points to affect. It focuses on performative presentation, relation, and interaction (via language) in interviews with couples and parenting a child with disabilities (Tröndle, 2022a). The position of parents of a child marked as disabled is of specific interest. It is potentially attached to ‘both sides’ of an othering along disability as a line of difference. Parents can (in a nearly forced way) play a role in the othering of their child. At the same time, they themselves become othered along disabling attributions in their position as ‘such parents’ (Ryan and Runswick-Cole, 2008; McLaughlin et al., 2008). Disability is thus understood as—on the one hand—connected to experienced barriers and discrimination within the social-material structure of modern, industrialized societies. On the other hand, it is an attribution, appearing against the background of idealized concepts of bodies and abilities, which are associated with suffering, dependency, and need for acceptance. Both analytical levels are considered equally relevant and interdependent. For the case of couples parenting a child marked as disabled, I will foremost refer to disabling affect as the affection along with attributions to disability: The affective repertoire (see also: von Poser et al., 2019; Wechuli, 2023a) is attached to disability markers. From disability studies, we know this affect ranges from the suggestion of “suffering” (Payton and Thoits, 2011; Maskos, 2015).

“Shame,” Marks (1999) as resonance to the relational counterpart of the other’s affection, like “pity,” “fear,” or “disgust” (Hughes, 2012). And as its (if available) resisting equivalent, disability pride and celebration of diversity. Only a few studies have specifically addressed the emotional distress of parents of a child with a disability. For example, Jackson (2021) examined the emotional lives of fathers of children with disabilities. Lassinantti and Almqvist (2021) elaborated on gender expectations and pressures to possess certain cognitive skills, which are linked to diagnostic discourses. In addition, they refer to the concept of emotional responsibility (Doucet, 2001, 2015) as a concept related to gender equality. Kwok and Kwok (2020) discuss the emotional work of parents of children with autism in Hong Kong, and Courcy and Des Rivières (2017) elaborate on mother blaming experienced by mothers of children with autism spectrum disorder. Gray (2002) discussed felt and enacted stigma among parents. In short, with the exception of a greater emphasis on blame, these studies discuss quite similar affective repertoires to those of disabling affect, as far as the limited research on the topic can be said to indicate.

### 1.2 Subjectivation as ‘such’ a subject

The concept of subjectivation has gained importance in social sciences, philosophy, and educational science, and it relates to different theoretical traditions (for an overview, see Traue and Pfahl, 2022). Subjectivation understood as a process of subject formation is close to the understanding that Judith Butler (1997) suggested by referring to Hegel, Nietzsche, and Freud as the subjection in “doubling back upon itself” (ibid. 22). She argues that “whether the doubling back upon itself is performed by primary longings, desire, or drives, it produces in each instance a psychic habit of self-beratement, one that is consolidated over time as conscience” (ibid.). From this perspective, the subject and its conscience are constituted by interpellations into a specific subjectivity. Discursive knowledge and symbolic order enable the subject to recognize itself as ‘such a subject,’ intelligible, depending on and related to others. From this perspective, the subject can be understood as constitutively social and relational (Donati, 2015). Within subjection, the subject becomes recognizable and able to recognize itself. In addition to Honneth’s sense of recognition as valuation in different social spheres (Honneth, 1995), this is also meant as being seen as such, becoming addressable as an intelligible subject. Through subjectivation, the individual becomes able to act, to experience, and—of particular interest here—to be affected. This is, according to the ‘doubling back upon itself,’ part of the constitutive rejection of what is not part of the subject’s formation and therefore not available as conscience, or a loss to be mourned: “Is there not a longing to grieve—and, equivalently, an inability to grieve—that which one never was able to love, a love that falls short of the ‘conditions of existence’” (Butler, 1997, p. 24). From this perspective, affect is not located in emotional space that can be understood as chosen. The internalization of cultural norms creates interior space; it “fabricates the distinction between interior and exterior life, offering us a distinction between the psychic and the social that differs significantly from an account of the psychic internalization of norms” (Butler, 1997, p. 19). Accordingly, affection is embedded in this understanding of subject formation but is open to a collective transformation of discursive knowledge and an iteration of norms (Butler, 1990, 1993). As argued before, this is not understood as a simple “mirroring” (Traue and Pfahl, 2022) of ‘obligations to feel,’ but also an *activity* to bring about a turn toward the subjecting call (Althusser, 1977) or the iteration. A growing body of literature was developed in German-speaking social and educational science in about the last 15 years to reconstruct processes of subjectivation as empirical phenomena (e.g., Bosančić et al., 2022; Traue and Pfahl, 2022; Bosančić and Keller, 2019; Geimer et al., 2019; Bosančić et al., 2019; Spies and Tuider, 2017; Pfahl et al., 2015; Alkemeyer et al., 2013; Schürmann, 2013; Reh and Rabenstein, 2012; Traue and Pfahl, 2012; Pfahl, 2011; Spies, 2010; Traue, 2010). The empirical study on which this study reports is located in this field of empirical research on subjectivation and methodologically refers to biographical and interpretive methods within the scope of the sociology of knowledge.

## 2 Couple narration as affective interaction

The reported empirical study on couples parenting a child with disabilities is based on a qualitative research design with the interpretive analysis of 15 narrative biographical in-depth interviews with five heterosexual couples in Germany (Tröndle, 2022a). An initial interview with each partner on their story as a couple was followed by an individual interview with each partner on their respective life stories. This dataset was also used in other sociological studies on couples and work-sharing arrangements. It enables the reconstruction of complex couple arrangements by contrasting the co-constructed couple narration with the individual ‘stand-up-narration’ of each partner (e.g., Wimbauer and Motakef, 2017; Wimbauer, 2012). Field access was made via parent organizations and led by the search for couples who described themselves as parents of a child with disabilities, without focusing on specific impairments. This study focused on the ‘accepted social attribution’ of being parents of a child with disabilities. The interviews were supplemented by a questionnaire on biographical information, diagnoses, and support for health care. Due to the focus on work-sharing arrangements, the dataset includes only couples with dual-employment. Between 2014 and 2018, 15 interviews were conducted over 1–4 h and were fully transcribed by the author. The case presented in this study is based on the level of *couples* in focusing on work-sharing arrangements. The sample is relatively homogenous in terms of lived sexual orientation, the lack of international mobility, as well as with regard to the stability as a couple (no explicit stories of separation), and in their romantic and biological framing of parenthood (no co-parenting, adoption, etc.). The sample is heterogeneous in terms of place of residence and local infrastructure (urban, rural), as well as in terms of educational background, diagnoses of children, and the level of daily use of care support.^1^ The survey was conducted as part of a dissertation at the Humboldt University of Berlin and was conducted in accordance with the applicable ethical considerations of the university as well as with the Code of Ethics of the German Sociological Association (DGS)^2^ (e.g., informed consent, critical review of necessary data, and data protection). Regarding the German research context, Germany has a differentiated welfare state system to support families with a child with disabilities. However, it is characterized by a high level of segregation, which is vehemently defended, especially in the education system (Biermann, 2021; Powell et al., 2021). Furthermore, in the German context, significant differences remain between West and East Germany (old and new Länder). On the one hand, incomes are still comparatively higher in western Germany; there in eastern Germany, there are more extensive childcare structures, since in the former GDR, dual incomes were the norm for both partners. These structural conditions also affect how couples choose to share work and care. Consequently, the sample encompasses couples from both geographical regions. To ensure anonymity, all sensitive data was pseudonymized. The sequential analysis of the extensive narrations was conducted in collaboration with different interdisciplinary interpretation groups of researchers and structured as a successive process of theoretical sampling (Glaser and Strauss, 2017). The analysis is based on interpretive and biographical methods (Rosenthal, 2018; Denzin, 1989; Akremi et al., 2018), focusing on the content and interaction patterns of abduction and narration. Thus, it is based on methodologies derived from phenomenology and the sociology of knowledge and is guided by the hermeneutic interpretation of experience and interaction (Schütz, 1972). Specifically, the enacted interaction during the couple interview shows negotiations within the couple *in situ* and is, however, particularly suitable for approaching affective expression. Such negotiations become visible in occupying or staying silent about topics, in interrupting, and in expressing affection or marking it as not belonging to oneself, as only others feel. These practices of affective interaction are related to stories about and by the couples on the level of content. The empirically based theorization of the analysis finally suggests an understanding of the couple’s (also affective) arrangements as subjected as ‘special parents’ along the lines of gender and disability. The results were also related to historical discourses on parents of a child with disabilities in pedagogy and special education to shed light on institutionalized knowledge, becoming part of their presentation as parents. Thus, the reconstruction of (disabling) affects, in the case of this study, is based on narration and the interaction in narration. In addition to the presentation and interpretation of the couple, the embedding of interactively performed activity in narration was interpreted in terms of discursive knowledge.

## 3 Subjectivation as ‘special parents’

The study revealed that, on the level of narrative structure (what kind of story has been told and how), the couples presented themselves from the position of ‘special parents.’ This is not trivial at all if we consider that the interview was about *their story as a couple* and that the aspect of dual employment was as much part of the sample strategy as the aspect of being parents of a child with disabilities. Without space to present the whole picture here, this was interpreted as a quasi-unavoidable formation along the discursive form of ‘special parenthood’ within excluding and ableist structures of society. Couples find themselves constantly addressed as a specific other, as ‘special parents,’ and as not fitting into the expectation of parenthood. This happens in everyday life as well as in education, organizations, and medical health care. Experiences of othering include all the ableist reactions we know from disability studies, such as pity, avoidance, aggression, staring, and exclusion. Being constantly confronted with othering is also associated with professionals in medical and specialized health care who are considered co-therapists and specialists for their child. The experience of a subject position as ‘special parents’ is thus twofold: exclusion via othering and discrimination on the one hand and acknowledgment of a special expertise on their child on the other hand. The latter includes being pushed toward an othering of their own child. This approach has been criticized by disability studies and led to the positioning of parents as “part of the problem” (Goodley and McLaughlin, 2008, p. 6). At the same time, this positioning excludes parents from subversive and empowering positions as allies for their child, connected to pride and anti-oppressive practices (Tröndle et al., 2024; Carey, 2020; Ryan and Runswick-Cole, 2008). Disability movements are critical to parental perspectives because of power imbalances in care relationships. Additionally, processes of subjectivation urge them into ‘special parenthood,’ including involvement in segregating practices. The couples learn to identify with ‘special parenthood,’ although it comes along with othering and discrimination (Tröndle, 2022a). Additionally, this subjectivation as ‘such a subject’ concerns not a single subject but a collective (parental) subject (Tröndle, 2022b). However, how does this subjectivation shape affect? How does it become felt to be ‘special parents?’ I address these questions with some illustrative empirical examples of negotiating affect in partnership.

## 4 Delegating the disabling affect in a partnership

The reconstruction of the interview data revealed that the disabling categorization of a child also shapes affection and specific forms of emotional self-understanding as its cognition. Mediating institutionalized structures of segregation and shapes of knowledge on disability, parents can hardly resist representations of themselves as suffering, accepting, and coping, or special. Within the reconstruction of couple narrations, a specific interactive practice of negotiating disabling affect appeared, which is illustrated by the following (anonymized) sequences. In one of the couple’s interviews, a woman (who is named here as Jannike Michaelis) is talking about a difficult situation after the birth of her daughter. Due to complications during birth, the child may develop an impairment. To clarify this in advance: The sequence is not chosen due to the narrated event but to illustrate the structural dynamic of this negation of affect in the couple, which becomes especially visible in that part of the interview. Mrs. Michaelis states about the experienced situation:

Mrs. Michaelis: *“[…] really, really hard, the biggest crises in my life (-) very terrifying, (---) I was in a state of emergency, helpless, powerless, (5) mh these are all characteristics and behaviors, which I had absolutely never known in my life before. […]”* (Interview with Ms. Michaelis and Mr. Löbe, translated).

The perspective from which this period is narrated is striking. The affective state presents as if the patient were completely alone. It is *her* crisis, *her* anxiety, and *her* feelings of great alarm and powerlessness. The phrase feeling “helpless” is also an explicit expression of being alone. Later in the interview, she explained her feelings of loneliness and feeling overwhelmed.

In the individual biographical interview, the male partner (who is called here Wolfgang Löbe) discusses his reaction to the same situation after the birth of his child:

Mr. Löbe: *“[…] yes (--) and (-) it was then, (-) well a shock, the birth was a shock, yeah it was like that. (--) and erm (--) I mean (-) I was not ready for this (---) yes, well I (-) withdrew myself inside somewhere (-) yeah, because I could not bear this. Hospital and (---) yeah, (--) well I know that Jannike [his wife] erm has not felt cared for by me, but I wasn’t able to do it differently yeah, I was escaping into getting things done, I would say, but then, to be there at her side at all times that wasn’t possible. […].”* (Interview with Mr. Löbe, translated).

The narration structure highlights Mr. Löbe’s difficulties in talking about the situation, his feelings, and his wife’s interpretation of being left alone. He breaks up sentences, stops several times, and seems to search for the right words. According to his framing of the event as a shock, and as he is talking about his inability to stay with his wife in the hospital, we can imagine that he also experienced a crisis. His stated strategy to deal with this “shock” was to back away and leave his partner unsupported. Mrs. Michaelis, in return, does not see the possibility of backing away from the overwhelming situation. As a woman, she was supposed to stay with the child in the hospital, despite her own needs. In this respect, both partners refer to a very common gendered framing of needs: the man refers to the woman’s need for support and his limitations in answering it. He does not mention his own psychological needs or those of the child. In return, the woman referred to her own needs and lack of support. The woman is expected to take care of the child, whereas the father is expected to take care of the woman. Simultaneously, gender-specific experiences regarding different types of physical involvement in childbirth should also be mentioned. Thus, the embodied and gendered affects are particularly ambiguous in this context. However, the experienced shock, performed in both narrations, takes on a very different connotation at the level of interpretation: On the one hand, we have an understanding of a fundamental crisis, that is, one’s own, an overwhelming affect that belongs to the female partner. Conversely, shock is characterized as a compulsion to maintain distance (for the male partner).

The patterns of coping and interpretation of emotional affection are influenced by gender dynamics. The affection becomes gendered in the framing of the answer, the emotion, and the cognitive recognition of the specific feeling. The “shock”—as they both call it—is evaluated as a specific feeling or rejection according to gender norms (guilt versus suffering). Referring to Ahmed (2010a): the affect “sticks the subject and the norm” together; the gendered calling becomes part of one’s own subjectivity—the affective aspect of subjection.

Additionally, “suffering” is also the most common emotional attribution to disability (e.g., Maskos, 2015; Hughes, 2012), which becomes relationally negotiated in the couple as the “female form” (Thomas, 1999) of affection. However, this gendered interpretation is ‘felt as one’s own.’ This contributes to an understanding of subjects’ affection as constituted by the rejection of what is “impossible to feel” (Butler, 1997) and by embodied norms that urge to feel specifically (Hufendiek, 2016; Ahmed, 2010c). They can be interpreted as impulses that create a turn toward interpellation (Althusser, 1977).

To provide further insights into how these feelings (as the interpretation of affection) are negotiated within the couple, I present another example from the same couple interview, where the partners discuss the woman and her therapeutic support:

Mrs. Michaelis: *“[…] I got myself a therapist, but not because I was sick, (-) or psychologically damaged (-) It was just that I had to find a way of dealing with this whole feeling of being overwhelmed and with the strain on the partnership.”*

Mr. Löbe: *“and you- but you were traumatized. (-) That is definitely something where a therapist can help.”*

Mrs. Michaelis: *“Yes! And that was necessary, but beyond that, psychosocial counseling would have been helpful like a lot of other things in order to get orientation on how to live with a disabled child […]”* (Interview with Mrs. Michaelis translated).

In this sequence, the couple negotiates the psychological needs of the female partner. She is described as “traumatized”—and it is not to discuss whether that was the case or not—but it is crucial that in this situation of enormous strain for both partners, she is named as the one who is traumatized, suffering, and in need of help. Her therapy was legitimized in two ways: to overcome trauma and to deal with challenges in their partnership. Later in the interview, the couple discusses how they have found ways, again with therapeutic support, to share their feelings as well as their responsibilities in care. Additionally, they end up working full-time and have arranged options to reduce their work hours, if necessary. However, this process lasts several years, with a lot of support and a high level of reflection and, in both states, discussions around work-sharing tasks, which are frequently initiated by the women.

This is only one of several examples of the analysis. It appears to be always the woman who is named as the one who suffers, is traumatized, or has psychological problems. The couples seem to agree on locating these sorts of experiences and feelings to the women, while the men’s own feelings are hardly even mentioned. This observation might not be solely applicable to couples experiencing ableism but becomes understandable as a more generalized gendered structure in romantic couples, which must be proven empirically. Nevertheless, the affective repertoire mobilized in the couples is an attribute of disability. That disability is associated with suffering, and psychological dilemmas are a common attribution, not only in everyday life. Within research on parents of children with disabilities in the field of special education, this became, at least until the mid-eighties, a generalized underlying assumption in research on a “family tragedy” (Risdal and Singer, 2004; Ferguson, 2002, 2001; Tröndle, 2022a, pp. 58–73). However, the act of disabling the ‘call to suffer’ is predominantly experienced by the mother. In the context of shared challenges faced by both partners, this suffering is often delegated to the female partner.

One possibility of framing this observation is to simply assume different strategies of coping with disability caused by traditional gender roles, as Hinze (1999 [1991]) suggests. However, I want to argue that it is more adequate to explain the observation through the lens of “emotional inequalities” (Illouz, 2012). Illouz discusses the term market patterns of romantic choices, arguing that they are related to gendered expectations of (not) expressing emotions. This would lead to a common form of gendered oppression in romantic partnerships that she calls ‘emotional inequalities.’ Although the concept is used in a different context, it is an adequate framing of what these couples perform: Disabling affects are negotiated in the couple as belonging to the female partner, while the male partner seems to identify them as not belonging to him. This is not a simple affective difference, but it can be addressed through coping strategies to overcome the disabling affects. These strategies varied across the sample, ranging from positive thinking, seeking therapeutic help, and developing skills and expertise to attending parent support groups. Mothers perform emotional tasks. The withdrawal strategy is not readily available to mothers because it relies on the other partner to assume responsibility for care work, domestic duties, and emotional engagement. The delegation of affect within the couple is connected to the readiness of both partners to care for and organize support for both partners. However, as explained above, the understanding used here does not aim at a purposeful and functional use of emotionality. Rather, it is interpreted as a gendered othering along the label of disability. Disabling emotional engagement on the part of mothers is thus a necessity, protected by the existential needs of care for a child and gendered delegations of responsibility, rather than a choice to fulfill. One may posit that the normative expectation of emotional restraint represents a form of emotional engagement assigned to fathers. In this manner, the avoidance of emotional involvement can be viewed as a form of emotional effort that is required in accordance with gender norms. However, from a pragmatic perspective, these gendered emotional demands at the level of action are intertwined with other forms of sustained care work and the recognition of care work in relation to paid work.

To give an example from another case, a couple of interviews with Mr. and Mrs. Huber illustrate that the expected burden and coping practice are in some cases also *explicitly* attributed to the mother. Mr. Huber states about his wife, after she mentioned that she had read about parents’ associations:

Mr. Huber: *“And then you also cheered up a bit more because you had a goal or an anchor for you, something to get involved <<Mrs. Huber: Mhm>>. That was quite good, I must say. (-) Otherwise, you would have fallen into another hole.”* (Interview with Mrs. and Mr. Huber).

The “hole” Mr. Huber mentions refers to an expected emotional state of depression of Mrs. Huber’s if she had not had this “anchor,” represented by her involvement in the parental organization. Mrs. Huber partially agrees or at least does not explicitly disagree with the interpretation of her spouse. The mother further becomes the one caring about ambivalence within ‘special parenthood,’ coming along with othering and discrimination and in treating *her* ‘suffering.’

In the context of this study, the empirical examples are illustrative of the specific phenomenon of co-produced emotional inequalities that occur alongside disabling expectations of fitting into a particular subject position as ‘special parents.’ These cases are part of a comprehensive case reconstruction that points to the same gender dynamics of delegating disabling affect in couples in different ways. The experience of being ‘othered’ and addressed as ‘special’ and the task of coping with it are in the analyzed cases mostly delegated to the female partner. In return, masculine attributes seem to enable the rejection of disabling affect. The mother becomes the one who feels the disabling interpellations and takes up the emotional burden of feeling and treating the disabling affect. Thus, it is an example of an affect that is entangled with intersecting markers of difference. This interplay becomes a part of subjects’ affects. This intersecting emotional and gendered task in intimate relationships has been highlighted in disability research regarding “psycho-emotional disablism” in sexual relationships (Liddiard, 2014; see also Thomas, 1999).

Nevertheless, the delegation of affect in partnership is not confined to the couple. Societal rejection of disability, ableism, othering, and segregation contributes to the need for individual solutions in couples. Moreover, medical care systems can stabilize unequal arrangements in partnership by addressing mothers as co-therapists and as responsible for the organization and coordination of assistance for their children. At the same time, women experience discrimination in such organizations. Several women in the study talk about how they are treated in hospitals, where the father is praised for his commitment, while the mother is treated as a source of irritation and disruption. Besides the affection within the couple, several interviewees also reported strong emotions from the side of relatives. Some mentioned that their parents regularly cried on the phone about their children’s disabilities and that they felt urged to comfort them. Mrs. Huber, for example, mentioned a phone call with her mother after she received a diagnosis for their son:

Mrs. Huber: “And then I went home and called my mother and said Julian has a disability, he is mentally disabled. And my mother cried a lot on the phone. And I thought, why is she crying? She has no RIGHT to cry! Because it’s not that BAD, it’s not, he’s not, he’s still our JULIAN, I thought all the time. Why are they all so sad? He stays the way he is. (--) Maybe because it was also my feeling, that my mother was now DISAPPOINTED (-) disappointed in ME.” (Interview with Mrs. Huber, translated).

Facing disabling affect after the diagnosis, this mother is confronted with signs that her child is now seen as ‘someone else,’ someone to be mourned. Strikingly, she interprets the grief of her mother as disappointment in herself. In such situations, parents, and mothers in particular, are once again asked to perform emotional effort for others, to overcome or accept the disabling affect (of others). According to Runswick-Cole (2013), mothers are asked to perform emotional engagement by “wearing it all with a smile.” Lassinantti and Almqvist (2021) also referred to the potential of using gender discourses to resist or negotiate gendered responsibilities in parenting. For example, Bamberg (2022) elaborated on the concept of “counter discourses.” These comments make us aware of “how subjects can ‘talk back’” (Bosančić et al., 2022).

## 5 Affecting disability as activity in subjectivation

This study explored gendered emotional dynamics in couples with one child classified as disabled. This study demonstrates that through subjectivation processes, mothers tend to take the emotional burden of ‘suffering,’ which is attributed to disability and special parenthood. Fathers constrained by gender-specific norms of affection tend to withdraw emotionally. This study further highlights how medical and social institutions reinforce these gendered roles. It has been argued that these patterns reflect and lead to ‘emotional inequalities’ in partnerships, whereby disabling emotions are delegated to the female partner, leading to other forms of emotional effort and care work. The delegation of disabling emotions in partnership as part of a specific subjectivation also points to a shared interest in disability studies and the sociology of emotions: approaching interrelations of materiality, bodies, social structure, and cultural frames of interpretation. In disability studies, these interrelations are often pursued with regard to questions of ableism, othering, and discrimination, but also in regard to the potential forgetting of bodies in the light of social models and strong emotions evoked by the “Non-Disabled Imaginary” (Hughes, 2012) toward disabled bodies (Hughes, 2012, 2009; Hughes and Paterson, 1997). For example, by asking about disabling (material and social) barriers to inclusion and how they are historically gained and interactively performed. In addition, the entanglements of reifying knowledge on disability with generalized and idealized concepts of ability and bodies are examined (Campbell, 2008; Goodley, 2014). In the sociology of emotions, these interrelations between materiality, bodies, and cultural frames of interpretation are primarily discussed as transmission, mediation, bodies, and forms of emotions and affect (e.g., Brennan, 2015; Brinkema, 2014; Anderson, 2014; Gregg and Seigworth, 2010). In more recent contributions, affect has been discussed as situated between cognition, bodily affection, and the culturally enabled affordances of affect (Hufendiek, 2018; Ahmed, 2010c). We already see several overlaps between cultural studies and the sociology of emotion, often with regard to questions of identity and emotion, and primarily from perspectives of feminist and gender studies (Pedwell and Whitehead, 2012; Ahmed, 2010b). Theorization of embodied subjects is also discussed in both fields (for disability studies, e.g., Marks, 1999; for the theory of emotions, e.g., Hufendiek, 2018, 2016, 2014; Fuchs, 2024). For the case of romantic relations, the conceptual framing “emotional inequalities” (Illouz, 2012) serves to create a deeper understanding of gendered and ableist affective interactions in the couple (on working families see also Hochschild, 2012 [1989]). The empirical example of couples parenting a child with disabilities touches (at least) two cultural forms, associated with a specific powerful suggestion of an “affective repertoire” (von Poser et al., 2019; Wechuli, 2023a): Romantic partnership, including parenting, is an idealized “promise of happiness” (Ahmed, 2010c) on the one hand, and ableist affects associated with disability, as a ‘promise of suffering and dependency,’ along idealized concepts of ability on the other (Maskos, 2015; Campbell, 2008; Goodley, 2014; Buchner et al., 2015). Both frames participate in subject formation and affective activities to turn toward the recognition as ‘special parents.’ The intersecting affective attributions that take part in the acceptance of mothers to be ‘special parents’ encompass ambivalence, othering, and discrimination (Tröndle, 2022a).

I also want to argue that *affection* can be understood as the *activity of affecting* disabling interpellation. Thus, I understand disabling *affection* as being evoked by ableist cultural norms and attributions and as an embodied activity. From this perspective, affectivity is not necessarily a passive experience. It is rather an affective (body-)movement, an engagement in opening up toward change and formation that involves the whole, embodied subject. Furthermore, this activity of affecting bridges the contradiction of standing ‘alone’ for an othered collective subject. The mother is urged to, but also ‘ready to affect’ the disabling interpellation, while the father is partly enabled to reject it—not feeling or delegating the affect. The disabling affect helps both partners adhere to the cultural framework of romantic partnership. The couple is addressed together as parents, but ‘affecting and enacting a special parent’ is especially performed, acknowledged, and ‘felt’ by the mother. This involves consequences for the readiness to deal with interpellation. The recognition of oneself as ‘suffering’ and ‘coping’ can become a form of compulsion, while its rejection is not available due to the involved gendered power dynamics in couples and the dependency of the child on care. This could be understood as a way of not jeopardizing the ‘promised happiness’ as a romantic couple and family. I further suggest understanding this as *affective activity*, a practice related to what Sally Haslanger calls “cultural technē” in order to “[…] organize information and coordinate action, thought, and affect […]” (Haslanger, 2021, p. 63). This broad understanding of “ideology” allows us to capture disabling affect as an activity of a subjectivated feeling that is informed and organized by “clusters of concepts, background assumptions, norms, heuristics, scripts, metaphors […]” (ibid.), which are to be reconstructed in their relevance for the respective affection. Besides the theoretical framing of affect ‘sticking to objects’ as a practice used via “cultural technē.” I want to argue that the theoretical frame of subjectivation as subjection (Butler, 1997) is helpful to grasp the embodiment of disabling interpellations as part of the formation of subjected affectivity. The “open-ended in-between-ness” (Gregg and Seigworth, 2010, p. 3) of affect, as well as an understanding of *affective activity in subjectivation,* challenges longstanding theoretical dualisms such as body and mind in disability studies and affect theory.

The study on couples parenting a child with disabilities shows how affective activity can be performed in couple arrangements: disabling affect, attached to the collective subject of ‘special parents,’ becomes negotiated, accepted, rejected, or delegated in partnership (Tröndle, 2022a). This suggestion provokes an engagement with *subjects’ affection as relational activity informed by cultural technē, becoming felt and enacted in subjectivation*.

## Data Availability

The datasets presented in this article are not readily available because sensitive raw data cannot be shared. Requests to access the datasets should be directed to judith.troendle@uni.lu.

## Footnotes

^1^More detailed information can be found in the table in the Appendix.

^2^ https://soziologie.de/fileadmin/user_upload/dokumente/Ethik-Kodex_2017-06-10.pdf

## Appendix: Anonymized core sample couples and core characteristics

**Table 1:**

Couple		Employment constellation	Children (age, disabilities, lives in the household)	Residence
Core sample	Förster	**Employment arrangement** At the time of the first and second interviews, they were practicing a supplemental income arrangement: Mrs. Förster is 40% employed; Mr. Förster is 100% employed. At the time of the third interview, they were practicing a breadwinner housewife arrangement: Mr. Förster is 100% employed; Mrs. Förster is 0% employed. **Education and field of employment** Mr. Förster has an academic education and works in a highly skilled technical job. Mrs. Förster has a high school diploma (German: Abitur) and works in a skilled job in the health sector.	Child 1 is 14 years old. It does not have a disability. Child 2 is 12 years old. From, has a complex disability and requires extensive day and night support and care.	Rural area near a larger city in West Germany
	Michaelis/Löbe	**Employment arrangement** Dual career arrangement: Mr. Löbe is 100% employed; Mrs. Michaelis is 100% employed. **Education and field of employment** Mrs. Michaelis has an academic education and a highly qualified job in the field of international cooperation; Mr. Löbe has an academic education and a highly qualified job in the field of law.	Child 1 of Mr. Löbe from a previous relationship is 22 years old. It does not have a disability. It lives with the mother most of the time and in the couple’s household on a daily basis. Child 2 is 5 years old and has a complex disability. It lives in the couple’s household and requires extensive support and care (day and night).	Medium-sized town in West Germany
	Huber	**Employment arrangement** Supplementary income/dual-income arrangement: Mrs. Huber is 50–65% employed and also supports her husband’s business; Mr. Huber is approximately 100% self-employed in his own company (depending on the order situation). **Education and field of employment** Mr. Huber has a high school diploma. He has an apprenticeship and is self-employed as a locksmith; Mrs. Huber has a secondary school diploma. She has an apprenticeship and works in administration.	Child 1 is 19 years old and has a chronic illness that is not acute (no need for support). It does not live in the household anymore. Child 2 is 18 years old and has a cognitive and mild physical disability. It lives in the couple’s household and has a slight need for support in everyday life.	Rural area in West Germany
	Balke	**Employment arrangement** Supplementary income arrangement: Mrs. Balke is 50% employed; Mr. Balke (over 100% employed, only at home at weekends). **Education and field of work:** Mr. Balke has an academic education and a highly qualified job in the technical field; Mrs. Balke has an academic education and a highly qualified job in the technical field.	Child 1 of Mrs. Balke from a previous relationship is 28 years old. It does not have a disability and is not living in the household. Child 2 of Mrs. Balke from a previous relationship is 26 years old and has a cognitive and physical disability. It lives in the couple’s household and has a slight need for support in everyday life. Child 3 is 7 years old and has physical disabilities. It needs support in everyday life and health monitoring day and night.	Small town in West Germany
	Winkler	**Employment arrangement** Dual Employment arrangement: Mrs. Winkler is 75% employed; Mr. Winkler is 100% employed. **Education and field of work:** Mr. Winkler has an academic education and a highly qualified position in the field of education; Mrs. Winkler has a high school diploma (German: Abitur), an apprenticeship, and a qualified position in the field of education.	Child 1 is 30 years old. It does not have a disability and does not live in the household anymore. Child 2 is 28 years old and has a cognitive and physical disability. It has a moderate need for support in everyday life.	Big city in East Germany

## References

[ref1] Ahmed S. (2010a). “1 Happy Objects” The affect theory reader. eds. Gregg M. Seigworth G. J. (New York, USA: Duke University Press), 29–51.

[ref2] Ahmed S. (2010b). Killing joy: feminism and the history of happiness. Signs J. Women Cult. Soc. 35, 571–594. doi: 10.1086/648513

[ref3] Ahmed S. (2010c). The promise of happiness. Durham; London: Duke University Press.

[ref4] Akremi L. Baur N. Knoblauch H. Traue B. (Eds.) (2018). *Handbuch interpretativ forschen*, *Grundlagentexte Methoden*. Weinheim: Beltz Juventa.

[ref5] Alkemeyer T. Budde G. Freist D. (Eds.) (2013). *Selbst-Bildungen: soziale und kulturelle Praktiken der Subjektivierung*, *Praktiken der Subjektivierung 1*. Bielefeld: Transcript.

[ref6] Althusser L. (1977). *Ideologie und ideologische Staatsapparate: Aufsätze zur marxististischen Theorie*. *Positionen 3*. Hamburg: VSA.

[ref7] Anderson B. (2014). Encountering affect: capacities, apparatuses, conditions. Surrey a.o.: Ashgate.

[ref8] Bamberg M. (2022). “Positioning the subject. Agency between master and counter” in Positioning the subject. Methodologien der Subjektivierungsforschung/methodologies of subjectivation research. eds. Bosančić S. Brodersen F. Pfahl L. Schürmann L. Spies T. Traue B. (Wiesbaden: Springer VS).

[ref10] Biermann J. (2021). “Der Einfluss Der UN-BRK Auf Inklusive Bildung in Nigeria Und Deutschland” in Handbuch Inklusion international/international handbook of inclusive education: Globale, Nationale und Lokale Perspektiven auf Inklusive Bildung/Global, national and local perspectives. eds. Köpfer A. Powell J. J. W. Zahnd R.. 1st ed (Opladen, Berlin, Toronto: Verlag Barbara Budrich), 167–178.

[ref11] Bosančić Saša Brodersen Folke Pfahl Lisa Schürmann Lena Spies Tina Traue Boris, eds. (2022). Following the subject. Grundlagen und Zugänge empirischer Subjektivierungsforschung. Wiesbaden: Springer VS.

[ref12] Bosančić S. Keller R. (Eds.) (2019). Diskursive Konstruktionen: Kritik, Materialität und Subjektivierung in der wissenssoziologischen Diskursforschung. Wiesbaden: Springer VS.

[ref13] Bosančić S. Pfahl L. Traue B. (2019). “Empirische Subjektivierungsanalyse: Entwicklung des Forschungsfeldes und methodische Maximen der Subjektivierungsforschung” in Diskursive Konstruktionen: Kritik, Materialität und Subjektivierung in der wissenssoziologischen Diskursforschung. eds. Bosančić S. Keller R. (Wiesbaden: Springer VS).

[ref14] Brennan T. (2015). The transmission of affect. Ithaka, London: Cornell University Press.

[ref15] Brinkema E. (2014). The forms of the affects. Duke: University Press.

[ref16] Buchner T. (2018). “Die Subjekte der Integration: Schule, Biographie und Behinderung” in Julius. ed. Verlag K. (Julius Klinkhardt: Bad Heilbrunn).

[ref17] Buchner T. Pfahl L. Traue B. (2015). Zur Kritik der Fähigkeiten: Ableism als neue Forschungsperspektive der Disability Studies und ihrer Partner_innen. Zeitschrift für Inklusion 2.

[ref18] Butler J. (1990). Gender trouble: Feminism and the subversion of identity. 1. publ. ed. Thinking gender. New York a.o.: Routledge.

[ref19] Butler J. (1993). Bodies that matter. New York: Routledge.

[ref20] Butler J. (1997). The psychic life of power. Theories in subjection. Stanford: University Press.

[ref21] Campbell F. K. (2008). Refusing able (ness): a preliminary conversation about ableism. M/C J. 11. doi: 10.5204/mcj.46

[ref23] Carey A. C. (2020). Allies and obstacles: disability activism and parents of children with disabilities. Philadelphia: Temple University Press.

[ref24] Carey A. C. Block P. Scotch R. K. (2020). Allies and obstacles: disability activism and parents of children with disabilites. Philadelphia; Rome; Tokyo: Temple University Press.

[ref25] Courcy I. Des Rivières C. (2017). “From cause to cure”: a qualitative study on contemporary forms of mother blaming experienced by mothers of young children with autism spectrum disorder. J. Fam. Soc. Work. 20, 233–250. doi: 10.1080/10522158.2017.1292184

[ref26] Czedik S. Pfahl L. (2020). Aktivierende Arbeitsmarktpolitiken und berufliche Rehabilitation. Gouvernementalitätskritische Überlegungen zu Organisation, Funktion und Beschäftigungsbedingungen von Werkstätten für behinderte Menschen. Vierteljahresschrift für Heilpädagogik und ihre Nachbargebiete 89, 80–92. doi: 10.2378/vhn2020.art11d

[ref27] Denzin N. K. (1989). “Interpretive biography” in Qualitative research methods 17 (Newbury Park a.o.: Sage).

[ref28] Donati P. (2015) in The relational subject. ed. Archer M. S. (Cambridge: Cambridge University Press).

[ref29] Doucet A. (2001). ‘You see the need perhaps more clearly than I have’: exploring gendered processes of domestic responsibility. J. Fam. Issues 22, 328–357. doi: 10.1177/019251301022003004

[ref30] Doucet A. (2015). Parental responsibilities: dilemmas of measurement and gender equality. J. Marriage Fam. 77:224–242. doi: 10.1111/jomf.12148, PMID: 39910408

[ref31] Ferguson P. M. (2001). “Mapping the family: disability studies and the exploration of parental response to disability” in Handbook of disability studies. eds. Albrecht G. L. Seelman K. D. Bury M. (Thousand Oaks [u.a.]: Sage), 373–395.

[ref32] Ferguson P. M. (2002). A place in the familiy: an historical interpretation on parental reactions to having a child with a disability. J. Spec. Educ. 36, 124–131. doi: 10.1177/00224669020360030201

[ref33] Foucault M. (1989). *Surveiller et punir: naissance de la prison*. *Bibliothèque des histoires 0007883*. Paris: Gallimard.

[ref34] Fuchs T. (2024). Verkörperte Gefühle. Zur Phänomenologie von Affektivität und Interaffektivität. Berlin: Suhrkamp.

[ref35] Geimer A. Amling S. Bosančić S. (Eds.) (2019). Subjekt und Subjektivierung: empirische und theoretische Perspektiven auf Subjektivierungsprozesse. Wiesbaden: Springer.

[ref36] Glaser B. G. Strauss A. L. (2017). Discovery of grounded theory: strategies for qualitative research. 1st Edn. London: Taylor and Francis.

[ref37] Goodley D. (2014). Dis/ability studies: theorising disablism and ableism. 1st Edn. London [u.a.]: Routledge.

[ref38] Goodley D. Liddiard K. Runswick-Cole K. (2018). Feeling disability: theories of affect and critical disability studies. Disabil. Soc. 33, 197–217. doi: 10.1080/09687599.2017.1402752

[ref39] Goodley D. McLaughlin J. (2008). “Theorising parents, professionals and disabled babies” in Families raising disabled children. Enabling care and social justice. eds. McLaughlin J. Goodley D. Clavering E. Fisher P. (Houndmills, Basingstoke, Hampshire, New York: Palgrave Macmillan), 1–20.

[ref40] Gray D. E. (2002). ‘Everybody just freezes. Everybody is just embarrassed’: felt and enacted stigma among parents of children with high functioning autism. Sociol. Health Illn. 24, 734–749. doi: 10.1111/1467-9566.00316

[ref41] Gregg M. Seigworth G. J. (2010). “An inventory of shimmers” in The affect theory reader. eds. Gregg M. Seigworth G. J. (Durham: Duke University Press), 1–25.

[ref42] Haslanger S. (2021). Ideology in practice: what does ideology do? Milwaukee, Wisconsin: Marquette University Press.

[ref43] Hinze D. (1999 [1991]). Väter und Mütter behinderter Kinder: der Prozeß der Auseinandersetzung im Vergleich. Heidelberg: Winter.

[ref44] Hochschild A. R. (1979). Emotion work, feeling rules, and social structure. Am. J. Sociol. 85, 551–575. doi: 10.1086/227049

[ref45] Hochschild A. R. (2012 [1989]). The second shift. Working families and the revolution at home. New York: Penguin Books.

[ref46] Hochschild A. R. (2012 [1983]). The managed heart. Commercialization of human feeling. Berkeley/Los Angeles: University of California Press.

[ref47] Honneth A. (1995). The struggle for recognition: the moral grammar of social conflicts. Cambridge: Polity Press.

[ref48] Hufendiek R. (2014). “Whereabouts: locating emotions between body, mind, and world” in Rethinking emotion: interiority and exteriority in premodern, modern, and contemporary thought. eds. Campe R. Weber J., Interdisciplinary German cultural studies, vol. 15 (Berlin: De Gruyter), 351–379.

[ref49] Hufendiek R. (2016). “Embodied emotions: a naturalist approach to a normative phenomenon” in Routledge studies in contemporary philosophy 76. 1st ed (New York and London: Routledge Taylor & Francis Group).

[ref50] Hufendiek R. (2018). Explaining embodied emotions - with and without representations. Philos. Explor. 21, 319–331. doi: 10.1080/13869795.2018.1477985

[ref51] Hughes B. (2009). Wounded/monstrous/abject: a critique of the disabled body in the sociological imaginary. Disabil. Soc. 24, 399–410. doi: 10.1080/09687590902876144

[ref52] Hughes B. (2012). “Fear, pity and disgust: emotions and the non-disabled imaginary” in Handbook of disability studies. eds. Watson N. Roulstone A. Thomas C. (London: Routledge), 67–78.

[ref53] Hughes B. Paterson K. (1997). The social model of disability and the disappearing body: towards a sociology of impairment. Disabil. Soc. 12, 325–340. doi: 10.1080/09687599727209

[ref54] Illouz E. (2007). *Cold intimacies: the making of emotional capitalism*. *Making of emotional capitalism*. Cambridge, UK: Polity Press.

[ref55] Illouz E. (2008). *Saving the modern soul: therapy, emotions, and the culture of self-help*. *Culture of self-help*. Berkeley: University of California Press.

[ref56] Illouz E. (2012). Why love hurts: a sociological explanation. 1. Publ. reprinted Edn. Cambridge: Polity Press.

[ref57] Jackson A. J. (2021). Worlds of care: the emotional lives of fathers caring for children with disabilities. California series in public anthropology. Berkeley: University of California Press.

[ref58] Karim S. (2021). *Arbeit und Behinderung: Praktiken der Subjektivierung in Werkstätten und Inklusionsbetrieben*. *Disability Studies. Körper - Macht - Differenz; 16*. Bielefeld: Transcript Verlag.

[ref59] Kwok K. Kwok D. K. (2020). More than comfort and discomfort: emotion work of parenting children with autism in Hong Kong. Child Youth Serv. Rev. 118:105456. doi: 10.1016/j.childyouth.2020.105456

[ref60] Lassinantti K. Almqvist A.-L. (2021). “‘Mums are mums’. Negotiations of parenthood ideals among Swedish mothers with ADHD” in Close relations - family, kinship, and beyond. eds. Henriksson H. W. Goedecke K., Crossroads of knowledge (Singapore: Springer Nature), 193–208.

[ref61] Liddiard K. (2014). The work of disabled identities in intimate relationships. Disabil. Soc. 29, 115–128. doi: 10.1080/09687599.2013.776486

[ref62] Marks D. (1999). Disability: controversial debates and psychosocial perspectives. London: Routledge.

[ref64] Maskos R. (2015). Ableism und das Ideal des Autonomen Fähig-Seins in der kapitalistischen Gesellschaft. Zeitschrift für Inklusion 2.

[ref65] McLaughlin J. Goodley D. Clavering E. Fisher P. (Eds.) (2008). Families raising disabled children. Enabling care and social justice. Houndmills, Basingstoke, Hampshire, New York: Palgrave Macmillan.

[ref66] Meißner H. (2010). *Jenseits des autonomen Subjekts: zur gesellschaftlichen Konstitution von Handlungsfähigkeit im Anschluss an Butler, Foucault und Marx*. *Gender studies*. Bielefeld: Transcript.

[ref67] Mik-Meyer N. (2016). Othering, ableism and disability: a discursive analysis of co-workers’ construction of colleagues with visible impairments. Hum. Relat. 69, 1341–1363.

[ref68] Oliver M. (2009). Understanding disability: from theory to practice. 2nd Edn. Basingstoke [u.a.]: Palgrave Macmillan.

[ref69] Payton A. R. Thoits P. A. (2011). Medicalization, direct-to-consumer advertising, and mental illness stigma. Soc. Ment. Health 1, 55–70. doi: 10.1177/2156869310397959

[ref70] Pedwell C. Whitehead A. (2012). Affecting feminism: questions of feeling in feminist theory. Fem. Theory 13, 115–129. doi: 10.1177/1464700112442635

[ref71] Pfahl L. (2011). Techniken der Behinderung: der deutsche Lernbehinderungsdiskurs, die Sonderschule und ihre Auswirkungen auf Bildungsbiografien. Disability Studies 7. Bielefeld: Transcript.

[ref72] Pfahl L. Schürmann L. Traue B. (2015). “Das Fleisch der Diskurse. Zur Verbindung von Biographie- und Diskursforschung in der wissenssoziologischen Subjektivierungsanalyse am Beispiel der Behindertenpädagogik” in Erziehungswissenschaftliche Diskursforschung: Empirische Analysen zu Bildungs- und Erziehungsverhältnissen. eds. Fegter S. Kessl F. Langer A. Ott M. Rothe D. Wrana D. (Wiesbaden: Springer VS).

[ref73] Powell J. J. W. Pfahl L. Blanck J. M. (2021). “Sonderpädagogische Fördersysteme und inklusive Bildung” in Handbuch Bildungs- und Erziehungssoziologie. eds. Bauer U. Bittlingmayer U. H. Scherr A. (Wiesbaden: Springer VS).

[ref74] Reh S. Rabenstein K. (2012). “Normen der Anerkennbarkeit in pädagogischen Ordnungen. Empirische Explorationen zur Norm der Selbständigkeit” in Judith Butler: Pädagogische Lektüren. eds. Ricken N. Balzer N. (Wiesbaden: Springer VS).

[ref75] Ricken N. (2013). “Anerkennung als Adressierung. Über die Bedeutung von Anerkennung für Subjektivationsprozesse” in Selbst-Bildungen. Soziale und kulturelle Praktiken der Subjektivierung. eds. Alkemeyer T. Budde G. Freist D. (Bielefeld: Transcript).

[ref76] Risdal D. Singer G. H. S. (2004). Marital adjustment in parents of children with disabilities: a historical review and meta-analysis. Res. Pract. Pers. Severe Disabil. 29, 95–103. doi: 10.2511/rpsd.29.2.95

[ref77] Rosenthal G. (2018). Interpretive social research. An introduction. Göttingen: University Press.

[ref78] Runswick-Cole K. (2013). “‘Wearing it all with a smile:’ emotional labour in the lives of mothers of disabled children” in Disabled children’s childhood studies: critical approaches in a global context. eds. Curran T. Runswick-Cole K. (Basingstoke: Palgrave MacMillan), 105–118.

[ref79] Ryan S. Runswick-Cole K. (2008). Repositioning mothers: mothers, disabled children and disability studies. Disabil. Soc. 23, 199–210. doi: 10.1080/09687590801953937

[ref80] Schürmann L. (2013). Schmutz als Beruf: Prekarisierung, Klasse und Geschlecht in der Reinigungsbranche; eine wissenssoziologische Untersuchung. Arbeit, Demokratie, Geschlecht 17. Münster: Westfälisches Dampfboot.

[ref9001] Schütz A. (1972). The phenomenology of the social world. Evanston, Ill.: Northwestern University Press.

[ref81] Snyder S. L. Mitchell D. T. (2006). Cultural locations of disability. Chicago [u.a.]: University of Chicago Press.

[ref82] Spies T. (2010). Migration und Männlichkeit: Biographien junger Straffälliger im Diskurs. Bielefeld: Transcript.

[ref83] Spies T. Tuider E. (Eds.) (2017). Biographie und Diskurs: methodisches Vorgehen und Methodologische Verbindungen. Wiesbaden: Springer VS.

[ref84] Thomas C. (1999). *Female forms: experiencing and understanding disability*. *Disability, human rights and society*. Buckingham [u.a.]: Open University Press.

[ref85] Traue B. (2010). Das Subjekt der Beratung: Zur Soziologie einer Psycho-Technik. Bielefeld: Transcript.

[ref86] Traue B. Pfahl L. (2012). “Desubjektivierungen. Zum Verhältnis von Befähigung, Wissen und Recht nach dem Neoliberalismus” in Wechselverhältnis im Wohlfahrtsstaat. Dynamiken gesellschaftlicher Justierungsprozesse. eds. Bereswill M. Figlestahler C. Haller L. Y. Perels M. Zahradnik F. (Münster: Westfälisches Dampfboot).

[ref87] Traue B. Pfahl L. (2022). “What is subjectivation? Key concepts and proposals for future research” in Following the Subject. Grundlagen und Zugänge empirischer Subjektivierungsforschung. eds. Bosančić S. Brodersen F. Pfahl L. Schürmann L. Spies T. Traue B. (Wiesbaden: Springer VS).

[ref88] Traustadóttir R. (1991). Mothers who care: gender, disability, and family life. J. Familiy Iss. 12, 211–228. doi: 10.1177/019251391012002005

[ref89] Traustadóttir R. (1995). “A mother’s work is never done: constructing a “normal” family life” in The variety of community experience: qualitative studies of family and community life. eds. Taylor S. J. Bogdan R. Lutfiyya Z. M. (Baltimore: Paul H. Brookes), 47–66.

[ref90] Traustadóttir R. (2006). Disability und gender: introduction to the special issue. Scand. J. Disabil. Res. 8, 81–84. doi: 10.1080/15017410600831341

[ref91] Tröndle J. (2022a). Elternschaft als Othering: zur Subjektivation von Paaren als Eltern eines Kindes mit Behinderung. Wiesbaden: Springer VS.

[ref92] Tröndle J. (2022b). “‚Subjectivation à deux‘ – Paare als Kollektivsubjekt” in Positioning the subject: Methodologien der Subjektivierungsforschung/methodologies of Subjectivation research. eds. Bosančić S. Brodersen F. Pfahl L. Schürmann L. Spies T. Traue B. (Wiesbaden: Springer Fachmedien Wiesbaden), 261–285.

[ref93] Tröndle J. Pfahl L. Traue B. (2024). Beyond complicity or Allyship: towards a new understanding of caregivers. Res. Soc. Sci. Disabil. (RSSD) 15, 185–200. doi: 10.1108/S1479-354720240000015012

[ref94] von Poser A. Heyken E. Ta T. M. T. Hahn E. (2019). “Emotion repertoires” in Affective societies: key concepts. eds. Slaby J. von Scheve C. (Abingdon, Oxon: Routledge), 241–251.

[ref95] Waldschmidt A. (2017a). “Culture - theory - disability: encounters between disability studies and cultural studies” in Studies. Körper - Macht - Differenz. eds. Berressem H. Ingwersen M., vol. 10Disability (Bielefeld: Transcript).

[ref96] Waldschmidt A. (2017b). “Disability goes cultural: the cultural model of disability as an analytical tool” in Culture - theory - disability: encounters between disability studies and cultural studies. ed. Waldschmidt A., Disability Studies. Körper - Macht - Differenz (Bielefeld: Transcript), 19–27.

[ref97] Waldschmidt A. Schneider W. (2012). “(Nicht-) Behinderung anders denken” in Kultur: von den Cultural Studies bis zu den Visual Studies; eine Einführung. ed. Moebius S. (Bielefeld: Transcript), 128–150.

[ref98] Wechuli Y. (2023a). Medicalizing disabled people's emotions-symptom of a dis/ableist society. Front. Sociol. 8:1230361. doi: 10.3389/fsoc.2023.1230361, PMID: 38148881 PMC10750362

[ref99] Wechuli Y. (2023b). “Zwischen Cripping und Reclaiming – wie die Disability Studies strategisch mit Gefühlen umgehen” in Zeitschrift für Disability Studies 1/2023. (Innsbruck: Innsbruck university press, ZDS)

[ref100] Wechuli Y. (2024). “Behindernder Affekt – Auswirkungen von Emotionen auf Lebenswirklichkeiten aus Perspektive der Disability Studies” in Ambivalente Emotionen im Kontext von Inklusion und (Bad Heilbrunn: Bundesvereinigung Lebenshilfe, Verlag Julius Klinkhardt) Behinderung. eds. Dederich M. Schuppener S..

[ref101] Wimbauer C. (2012). Wenn Arbeit Liebe ersetzt: Doppelkarriere-Paare zwischen Anerkennung und Ungleichheit. Frankfurt [u.a.]: Campus.

[ref102] Wimbauer C. Motakef M. (2017). Das Paarinterview: Methodologie- Methode- Methodenpraxis. Wiesbaden: Springer VS.

